# Mixed Phenotype Acute Leukemia Presenting as Leukemia Cutis

**DOI:** 10.1155/2016/1298375

**Published:** 2016-11-10

**Authors:** Geetha Narayanan, M. T. Sugeeth, Lali V. Soman

**Affiliations:** Department of Medical Oncology, Regional Cancer Centre, Trivandrum 695011, India

## Abstract

Leukemia cutis (LC) is defined as infiltration of the skin by leukemic cells resulting in clinically recognizable cutaneous lesions. It is common in congenital leukemia and acute myeloid leukemia. However, LC has rarely been reported with mixed phenotypic acute leukemia (MPAL). We report the case of a lady who presented with erythematous papular and nodular lesions all over the body. Skin biopsy showed leukemic infiltration and bone marrow aspiration showed MPAL of the T/myeloid with monocytic differentiation lineage. This is the first report of an adult patient with MPAL of the T/myeloid with monocytic differentiation type presenting with leukemia cutis. She was started on chemotherapy with Hyper-CVAD. There is complete resolution of the skin lesions and she has achieved bone marrow remission after the first cycle of chemotherapy.

## 1. Introduction

Leukemia cutis (LC) is defined as infiltration of the skin by leukemic cells resulting in clinically recognizable cutaneous lesions [1]. It is one of the extramedullary manifestations of leukemia. LC can occur with any type of leukemia but is most common in congenital leukemia (25–30%) and acute myeloid leukemia (AML) (10–15%) and is less frequent with acute lymphoblastic leukemia (ALL) (1–3%) [2–4]. However, LC has rarely been reported with mixed phenotypic acute leukemias (MPAL). We report the case of a MPAL of the T/myeloid with monocytic differentiation lineage presenting with leukemia cutis.

## 2. Case Report

A 40-year-old lady presented with history of a reddish painless swelling in the left forearm since 3 months, gradually increasing in size. During the following days, she developed multiple nodular swellings all over the body. There was no fever or weight loss. She presented to us following a biopsy from the forearm swelling.

**Figure 1:**
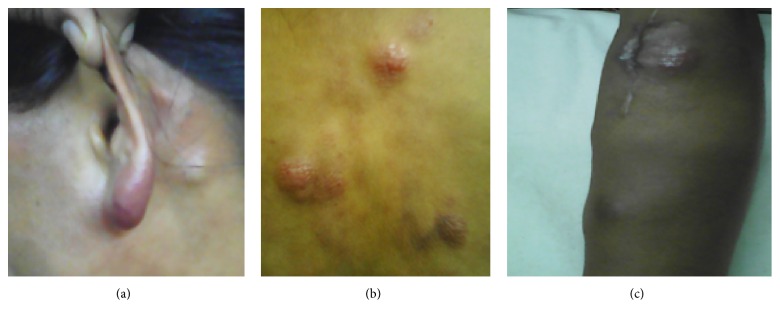
Images of the ear, back, and forearm showing multiple erythematous papular and nodular lesions.

On examination, her general condition was good. She had multiple erythematous papular lesions and nodular swellings of 1–3 cm over both legs, both arms, chest wall, abdomen, back, face, scalp, and ear lobules (Figures 1(a), 1(b), and 1(c)). She also had bilateral supraclavicular and left cervical lymphadenopathy of 1 cm. There was no organomegaly.

**Figure 2:**
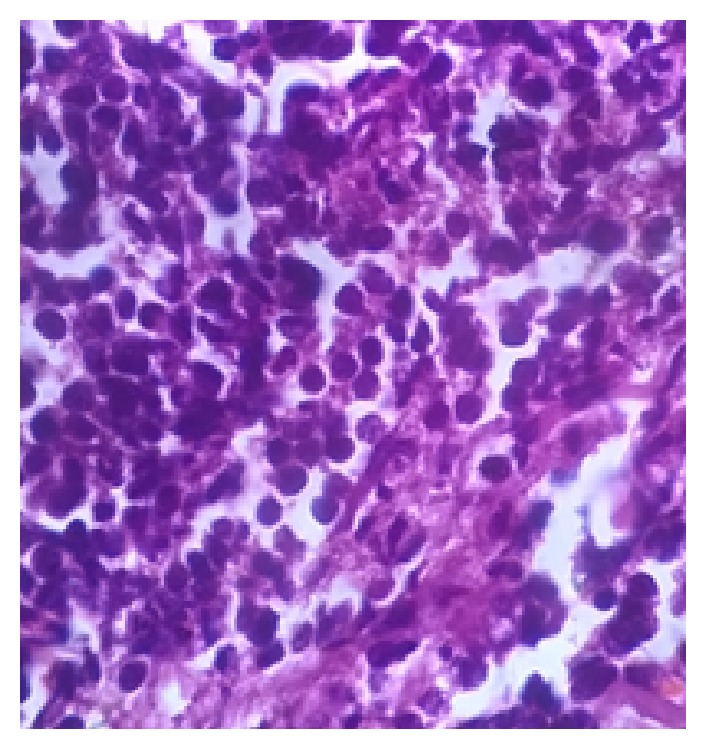
H7E ×100 Section from nodular skin lesion showing infiltration by leukemic cells.

**Figure 3:**
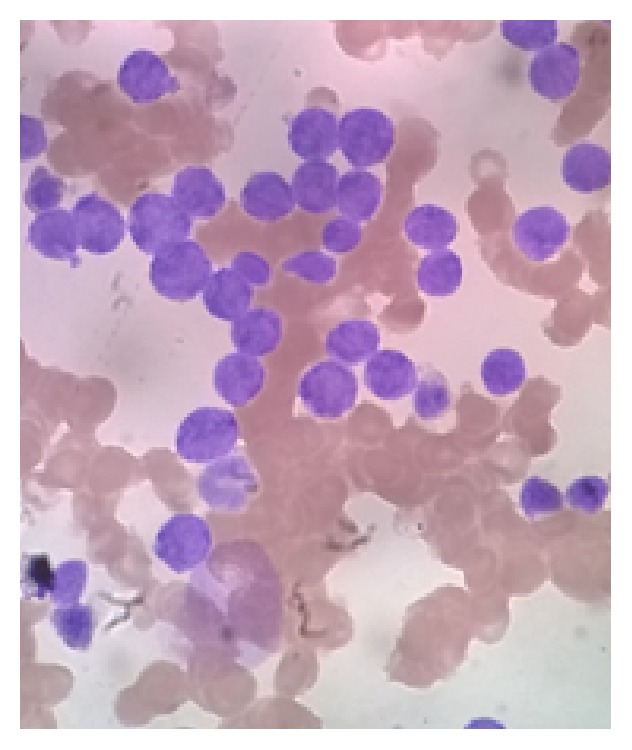
Bone marrow smear showing immature cells with blue cytoplasm, round nuclei, and nucleoli.

Her hemoglobin was 116 gm/L, total WBC count was 9.7 × 10^9^/L, platelet count was 236 × 10^9^/L, and the peripheral smear showed 42% blast cells. Her renal and liver functions were normal, and serum lactate dehydrogenase was 2327 U/L. Section from the skin biopsy showed diffusely arranged atypical cells, which were positive for LCA and negative for CD20 on immunohistochemistry (Figure 2). A bone marrow aspiration showed sheets of immature cells with blue vacuolated cytoplasm, round nuclei, and 1-2 nucleoli (Figure 3). The abnormal cells were positive for periodic acid schiff (PAS) and nonspecific esterase (NSE) and negative for peroxidase by cytochemistry. Immunophenotyping by flow cytometry showed the abnormal cells to be positive for the myeloid markers CD33 and CD11c and negative for the myeloid markers CD13, CD14, CD61, CD64, CD117, anti-MPO, and cyCD61. The cells were also positive for the T-cell markers cyCD3, CD4, CD5, and CD7 and negative for the T-cell markers CD3, CD8, CD56, anti-Tdt, TCrgd, TCrab, and CD1a. The cells were negative for all B-cell markers CD10, CD19, CD20, and cyCD79a. A diagnosis of mixed phenotypic acute leukemia of T/myeloid with monocytic differentiation lineage was made. The skin lesions showed good response to steroids. She was started on chemotherapy with Hyper-CVAD protocol, her skin lesions completely disappeared during the first cycle of chemotherapy, and she achieved complete bone marrow remission with <3% blasts after the first cycle. She has completed 2A cycle and continues to be in remission.

## 3. Discussion

MPAL is an uncommon disease that comprises 2–5% of all leukemias [5, 6]. MPAL can be classified as B/myeloid, T/myeloid, B/T lymphoid, or rarely trilineage leukemia [7]. The T/myeloid represents 35% of all MPAL [8]. Cases of MPAL of the T/myeloid with monocytic differentiation lineage are extremely rare. Our patient was diagnosed as T/myeloid with monocytic differentiation in view of the cyCD3, esterase, and CD11c positivity.

In leukemia cutis, there is infiltration of neoplastic leucocytes or their precursors into the epidermis, dermis, or subcutis, resulting in clinically detectable skin lesions. LC often occurs concomitantly with systemic leukemia but may also occur following the diagnosis or at the time of relapse. Rarely it occurs prior to the diagnosis of acute leukemia in blood or bone marrow when it is known as aleukemic leukemia [9].

LC displays a variety of clinical appearances. It can present as violaceous, erythematous, or hemorrhagic nodules, papules, vesicles, bullae, or plaques [1, 10]. The skin lesions are usually located in extremities and trunk; rarely they can occur at sites of herpetic lesions, trauma, intravenous catheters, recent surgical sites, and so forth [2, 3, 11]. Our patient also had erythematous papules and nodular skin lesions which on biopsy proved to be leukemic infiltrates.

LC is most commonly associated with AML especially M4 and M5 and congenital leukemia and occurs rarely in ALL [2, 3]. However, LC has not been described with adult MPAL; only a rare case of congenital MPAL associated with skin lesion is reported [12]. Our patient had MPAL of the T/myeloid with monocytic differentiation lineage. This is the first report of leukemia cutis as a presenting feature in an adult with MPAL of the T/myeloid with monocytic differentiation lineage.

Leukemia cutis is associated with certain genetic abnormalities such as t(8:21), inversion 16 [13, 14]. The mechanism behind the specific migration of leukemic cells to the skin is not clear. It has been speculated that the chemokine, integrin, and other adhesion (ICAM-1) molecules may play a role in skin specific homing of leukemic cells.

The treatment of LC is aggressive systemic chemotherapy directed at eradicating the specific type of leukemic cells. Patients with LC often have other sites of extramedullary disease and tend to have a poor prognosis [10, 15]. Our patient is started on aggressive chemotherapy, her skin lesions completely resolved, and she achieved complete marrow remission after the first cycle of chemotherapy. Currently she has completed 2A cycle of Hyper-CVAD and continues to be in remission.

## References

[1] Wagner G., Fenchel K., Back W. (2012). Leukemia cutis: epidemiology, clinical presentation, and differential diagnosis. *Journal der Deutschen Dermatologischen Gesellschaft*.

[2] Cho-Vega J. H., Medeiros L. J., Prieto V. G., Vega F. (2008). Leukemia cutis. *American Journal of Clinical Pathology*.

[3] Lee J. I., Park H. J., Oh S. T., Lee J. Y., Cho B. K. (2009). A case of leukemia cutis at the site of a prior catheter insertion. *Annals of Dermatology*.

[4] Su W. P. D. (1994). Clinical, histopathologic, and immunohistochemical correlations in leukemia cutis. *Seminars in Dermatology*.

[5] Aribi A., Bueso-Ramos C., Estey E. (2007). Biphenotypic acute leukaemia: a case series. *British Journal of Haematology*.

[6] Rubnitz J. E., Onciu M., Pounds S. (2009). Acute mixed lineage leukemia in children: The experience of St Jude Children's Research Hospital. *Blood*.

[7] Colovic M., Colovic N., Jankovic G. (2012). Mixed phenotype acute leukemia of T/myeloid type with a prominent cellular heterogeneity and unique karyotypic aberration 45,XY, dic(11;17). *International Journal of Laboratory Hematology*.

[8] Matutes E., Pickl W. F., Veer M. V. (2011). Mixed-phenotype acute leukemia: clinical and laboratory features and outcome in 100 patients defined according to the WHO 2008 classification. *Blood*.

[9] Yones S. S., Khorasgani M. G., Iqbal T. (2009). Aleukemic leukemia cutis. *Egyptian Dermatology Online Journal*.

[10] Kang Y. S., Kim H. S., Park H. J. (2013). Clinical characteristics of 75 patients with leukemia cutis. *Journal of Korean Medical Science*.

[11] Rao A. G., Danturty I. (2012). Leukemia cutis. *Indian Journal of Dermatology*.

[12] Ergin H., Özdemir Ö. M. A., Karaca A. (2015). A newborn with congenital mixed phenotype acute leukemia after in vitro fertilization. *Pediatrics and Neonatology*.

[13] Agis H., Weltermann A., Fonatsch C. (2002). A comparative study on demographic, hematological and cytogenetic findings and prognosis in acute myeloid leukemia with and without leukemia cutis. *Annals of Hematology*.

[14] Kubonishi I., Seto M., Murata N., Kamioka M., Taguchi H., Miyoshi I. (1998). Translocation (10;11)(p13;q13) and MLL gene rearrangement in a case of AML (M5a) with aggressive leukemia cutis. *Cancer Genetics and Cytogenetics*.

[15] Jang I. G., Lee D. W., Han C. W., Kim C. C., Cho B. K. (1996). A clinical observation on leukemia cutis. *Korean Journal of Dermatology*.

